# The key role of microtubules in hypoxia preconditioning-induced nuclear translocation of HIF-1α in rat cardiomyocytes

**DOI:** 10.7717/peerj.3662

**Published:** 2017-08-14

**Authors:** Hai Guo, Hong Zheng, Jianjiang Wu, Hai-ping Ma, Jin Yu, Maimaitili Yiliyaer

**Affiliations:** Department of Anesthesiology, The First Affiliated Hospital of Xinjiang Medical University, Urumqi, Xinjiang, China

**Keywords:** HIF-1α, Microtubules, Myocardial cells, Hypoxia preconditioning

## Abstract

**Background:**

Hypoxia-inducible factor (HIF)-1 is involved in the regulation of hypoxic preconditioning in cardiomyocytes. Under hypoxic conditions, HIF-1α accumulates and is translocated to the nucleus, where it forms an active complex with HIF-1β and activates transcription of approximately 60 kinds of hypoxia-adaptive genes. Microtubules are hollow tubular structures in the cell that maintain cellular morphology and that transport substances. This study attempted to clarify the role of microtubule structure in the endonuclear aggregation of HIF-1α following hypoxic preconditioning of cardiomyocytes.

**Methods:**

Primary rat cardiomyocytes were isolated and cultured. The cardiomyocyte culture system was used to establish a hypoxia model and a hypoxic preconditioning model. Interventions were performed on primary cardiomyocytes using a microtubule-depolymerizing agent and different concentrations of a microtubule stabilizer. The microtubule structure and the degree of HIF-1α nuclear aggregation were observed by confocal laser scanning microscopy. The expression of HIF-1α in the cytoplasm and nucleus was detected using Western blotting. Cardiomyocyte energy content, reflected by adenosine triphosphate/adenosine diphosphate (ATP/ADP), and key glycolytic enzymes were monitored by colorimetry and high-performance liquid chromatography (HPLC). Reactive oxygen species (ROS) production was also used to comprehensively assess whether microtubule stabilization can enhance the myocardial protective effect of hypoxic preconditioning.

**Results:**

During prolonged hypoxia, it was found that the destruction of the microtubule network structure of cardiomyocytes was gradually aggravated. After this preconditioning, an abundance of HIF-1α was clustered in the nucleus. When the microtubules were depolymerized and hypoxia pretreatment was performed, HIF-1α clustering occurred around the nucleus, and HIF-1α nuclear expression was low. The levels of key glycolytic enzymes were significantly higher in the microtubule stabilizer group than in the hypoxia group. Additionally, the levels of lactate dehydrogenase and ROS were significantly lower in the microtubule stabilizer group than in the hypoxia group.

**Conclusion:**

The microtubules of cardiomyocytes may be involved in the process of HIF-1α endonuclear aggregation, helping to enhance the anti-hypoxic ability of cardiomyocytes.

## Introduction

The hypoxia-induced injury of cardiomyocyte is a major cause of preoperative and death in patients with coronary heart disease (Rossini et al., 2014). In 1986, Murry, Jennings & Reimer (1986) proposed the concept of hypoxic Preconditioning (HPC), namely, that repeated exposure to brief periods of hypoxic in advance can reduce subsequent cardiomyocyte injury caused by long-term ischemia. HPC has a close relationship with Hypoxia-inducible factor (HIF-1). HIF-1 is a nuclear transcription factor of cardiomyocytes that is expressed under hypoxic conditions (Shohet & Garcia, 2007). Hypoxia can activate many hypoxia adapted genes improve the anti-hypoxia ability of cardiomyocytes. The present study has clarified that HIF-1 is a heterodimer transcription factor consisting of α and β subunits (Tuomainen & Tavi, 2017). When hypoxia occurs, HIF-1α accumulates in the cytoplasm before being transferred to the nucleus and associating with β subunits to form the HIF-1αβ complex, thus activating downstream hypoxia responsive genes, such as erythropoietin (EPO), vascular endothelial growth factor (VEGF), and glucose transferase (Glut-1) (Tuomainen & Tavi, 2017). These hypoxia-responsive genes can all increase the blood supply to myocardial tissue, improve the energy metabolism after myocardial ischemia, reduce the myocardial infarction area, and protect the myocardium as a result (Jain et al., 2013; Kim, Rhim & Lee, 2011; Ong & Hausenloy, 2012). HIF-1α nuclear translocation is a key step in the activation of endogenous myocardial protection mechanisms by HIF-1. Unfortunately, the specific mechanism and factors influencing HIF-1α nuclear translocation have not yet been fully elucidated.

Microtubules are hollow tubular structures in the cell and are composed of 2 subunits: the α and β subunits. In addition, they are also involved in the formation of a variety of microtubule associated proteins (MAPs) that aid in microtubule function (Miragoli et al., 2016). Cardiomyocyte microtubules are distributed parallel to the distribution of muscle fibers and are wrapped around the nucleus (Kumarapeli & Wang, 2004). Additionally, they are distributed throughout the cytoplasm in the cardiomyocytes, with one end connected to the nucleus and the other end connected to the muscle cytoplasm (Robison & Prosser, 2017). These distribution characteristics play an important role in maintaining the normal morphology and position of the nucleus (Xiaodong et al., 2015). Microtubules also maintain cell morphology as an intracellular material transport pathway. It has been reported that many important transcription factors, such as p53, androgen receptor, thyroxin and glucocorticoid receptor β, may be directly combined with nerve cell microtubules and expressed prokaryotically throughout the microtubule structure (Jiang et al., 2015). Once the microtubule network structure is lost, the levels of key glycolytic factors also decrease significantly.

Studies have reported that damaged microtubule structures might lead to decreased expression of the HIF-1α protein and its associated downstream hypoxia-responsive proteins or signaling pathways in tumor cells and some hypoxic cells (Duyndam et al., 2007; Escuin, Kline & Giannakakou, 2005). Considering the above results, we proposed the following scientific hypotheses that cardiomyocyte microtubules may play a key role in the nuclear translocation of HIF-1α, and the stabilization of microtubule structure can enhance the resistance of myocardial HPCs to hypoxia injury. To clarify the above hypotheses, we cultivated primary cardiomyocytes, established a hypoxia injury model in cardiomyocytes through hypoxia preconditioning, and attempted to clarify the role of microtubule structure in HIF-1α nuclear translocation by observing the intracellular distribution of HIF-1α with immunofluorescence staining.

## Materials and Methods

### Primary cardiomyocyte culture

This research was performed in accordance with the guidelines formulated by the Animal Welfare Act of Xinjiang Medical University and the Care and Application Guideline of Experimental Animals of Xinjiang Medical University. The protocol was approved by the Committee on the Ethics of Animal Experiments of the First Affiliated Hospital of Xinjiang Medical University (Permit Number: IACUC-20130217059). Animals were housed in an environment with a constant temperature and light/dark cycles and with access to water and food. The male and female Sprague Dawley (SD) rats used in this study were given standard rodent chow.

### Separation of primary cardiomyocytes

SD rats were selected within 24 h of birth and were anesthetized with 5% of isoflurane and then subjected to cervical dislocation. After disinfection, rat hearts were removed via thoracotomy and immediately rinsed three times with precooled PBS, after which the atrium was removed. The rest of the ventricles were cut into 0.5 m3-sized blocks, which were placed into a centrifuge tube before 2 ml 0.125% trypsin was added. Then, the samples were digested in a water bath at 37 °C for 8 min. The supernatant was aspirated, and M199 culture medium containing 15% fetal bovine serum was added to terminate digestion. Two milliliters of 0.125% trypsin were added to the block and allowed to digest for 8 min, and then the supernatant was aspirated; this procedure was repeated until the block was digested and the cells were separated. The collected supernatant was centrifuged at 1,000 rpm for 10 min to collect the cells, which were then cultivated in a CO_2_ incubator (5% CO_2_, 95% air, 37 °C) for 1 h. Differential adhesion was used to remove fibroblasts, which were seeded into the corresponding culture plate. Then, cells were cultivated in a CO_2_ incubator (5% CO_2_, 95% air, 37 °C) (Malhotra et al., 2002; Yu et al., 2016; Zheng et al., 2015). The culture medium was changed every other day. BrdU (0.1 mM) was added two days in advance to inhibit the growth of fibroblast cells. The isolated cardiomyocytes were randomly grouped for experiments three days after processing.

### Hypoxic injury and preconditioning of primary myocytes and grouping

To induce hypoxia injury, the buffer (0.9 mM NaH_2_PO_4_, 6 mM NaHCO_3_, 1.8 mM CaCl_2_, 1.2 mM MgSO_4_, 20 mM, HEPES, 98 mM NaCl, 10 mM KCI, pH 6.8) was saturated with a gas mixture with low-oxygen concentration (94% N_2_, 5% CO_2_ and 1% O_2_) for 30 min, and then it was used to replace the normal M199/EBSS medium (HyClone, Logan, UT, USA) in the culture flasks. Cells were incubated in the low-oxygen buffer (MIC101; Billups-Rothenberg, San Diego, CA, USA) for 6 h, after which the low oxygen buffer was replaced with normal medium and the cells were incubated in a normal environment for 1 h. To produce HPC, the buffer described previously was saturated with a gas mixture with low-oxygen concentration for 30 min, and then used to replace the normal medium. Cells were incubated in the low-oxygen buffer for 1 h, and then switched to a normal medium and incubated in a normal environment for 1 h. This low/normal oxygen cycle was repeated three times (Zheng et al., 2015).

All experimental groups were placed in a CO_2_ incubator (5% CO_2_, 95% air, 37 °C) for 2 days and were then divided into the following groups: (1) N group: normal culture of cardiomyocytes that were not treated with hypoxia preconditioning, (2) H group: cardiomyocytes that were allowed to recover at normal oxygen levels after 6 h of hypoxia, (3) HPC group: cardiomyocytes that were treated with hypoxia injury after hypoxia preconditioning and were subjected to hypoxia injury for 6 h, (4) HT group: paclitaxel (Sigma, St. Louis, MO, USA) was added into the culture medium at final concentrations of 5 µmol/L (HT5), 10 µmol/L (HT10), or 15 µmol/L (HT15), and cells were exposed to hypoxia for 6 h, (5) HC group: colchicine (Sigma, St. Louis, MO, USA) was added into the culture medium at a final concentration of 10 µmol/L for 6 h, and then cells were exposed to hypoxia. HPC + HT10 group: cardiomyocytes that were treated with hypoxia injury for 6 h after hypoxia preconditioning and paclitaxel was added into the culture medium at final concentrations of 10 µmol/L. The number of cardiomyocytes were determined in each group according to the content of each test. In Result 1, each group was divided into nine samples. In Result 2, each group had nine samples. In Result 3, there were seven samples in each experimental group. In Result 4, each group had three samples. In Result 5, each experiment was divided into 12 samples.

Paclitaxel can stabilize the microtubule structure, so it was used to depolymerize microtubules. Colchicine is the depolymerizing agent for structure and is able to depolymerize microtubules while inhibiting cell proliferation and division (Teng et al., 2012); however, very few cardiomyocytes of new-born rats have the ability to divide. In addition, the proliferation of most cardiomyocytes of new-born rats only occurs within 48 h of birth. In our model, we added colchicine to all groups after 48 h, thereby removing the influence of colchicine on the cardiomyocyte cell cycle. In addition, we added BrdU to inhibit the cell division of fibroblasts; nevertheless, it had little effect on cardiomyocyte division.

### Immunofluorescence assay of cardiomyocyte microtubules and HIF-1α

We used an anti-rat α-microtubulin antibody (Thermo Fisher Scientific; Waltham, MA, USA) and an anti-rat HIF-1 antibody (Thermo Fisher Scientific; Waltham, MA, USA) as primary antibodies and FITC- and tetramethylrhodamineisothiocyanate (TRITC)-conjugated antibodies (Jackson Immuno-Research, West Grove, PA, USA) as secondary antibodyes. Nuclei were counterstained with 4′, 6-diamidino-2-phenylindole (DAPI; Biotium, Hayward, CA, USA). The experiment used an LSCM510 META laser confocal scaning microscope for observation and ImageJ software to measure and analyze the red and green fluorescence intensity of individual cells. The fluorescence intensity of each normoxic cell was set to a value of 1.0, and the hypoxia group was the comparison group. Cardiomyocytes were cultured on coverslips (10-mm diameter) and stained, and the coverslips were divided into five fields. One intact cardiomyocyte was randomly selected from each field for photography and analysis. Three cells were analyzed from each coverslip, and three coverslips were analyzed for each group; thus, nine cardiomyocytes were observed and analyzed for each group.

### Determination of ATP and ADP content

Perchloric acid and high performance liquid chromatography (HPLC) were used to determine ATP and ADP levels (Napolitano & Shain, 2005). Cardiomyocytes were added into 50% perchloric acid to lyse the cells; centrifugation was performed for the cell lysates under the conditions of 4 °C and 12,000 rpm for 30 min. The supernatant was transferred to a new centrifuge tube and 2.5 M K_2_CO_3_ was added, the pH was adjusted to 6.5, and the solution was centrifuged for 30 min under the same conditions. Then, the supernatant was immediately collected for analysis. High-performance liquid chromatography was used as the determination method. The detection conditions were as follows: the analytical column used was HYPERSIL Cl8 5u; the column length was 250 mm; the column diameter was 4.6 mm; the mobile phase was 0.005 mol/L pH 6.0 H_2_PO_4_; and the detector was UV 254 nm (H_2_PO_4_). The column speed was 0.9 ml/min; the sample volume was 20 ul; the analysis time lasted 10 min. The results were expressed as ug/g protein. To ensure the consistency, each experiment was run in duplicate, and each group was evaluated six times (*n* = 12).

### Determination of lactate dehydrogenase (LDH), hexokinase (HK), and pyruvate kinase (PK) levels in culture supernatants

PFK was measured based on the methods of (Napolitano & Shain, 2005). LDH, HK and PK were measured with an enzyme-linked immunosorbent assay, which was performed according to the instructions provided in the kit (Nanjing Jiancheng Biotech, Nanjing, China). The results were analyzed with a Beckman DC-7 ultraviolet-visible light spectrophotometer. Each experiment was repeated twice, and six samples were analyzed for each group (*n* = 12).

### Determination of HIF-1α protein expression

HIF-1α protein in the nucleus and cytoplasm was detected. Western blotting referenced to our previous research methods. Cardiomyocytes were rinsed with pre-cooled phosphate buffer saline (PBS) for 1–2 times. Myocardial nucleoprotein and cytoplasm proteins (KeyGen Company, Nanjing, China) were extracted according to the nucleoprotein and cytoplasm protein kits. After BCA method was used to measure protein concentration, protein loading buffer was added, and boiled in the boiling water for 5 min, and persevered in a refrigerator at −20 °C. Each group of myocardial protein samples was given electrophoresis, trans-membrane and blocking. After blocking, prepared primary antibody buffer, HIF-1a (1:1,000), and β-actin (1:1,000) were added and cultured at 4 °C overnight. Secondary antibody buffer (1:1,000) was added and cultured at room temperature for 2 h. Developer A and B were mixed and 1mL mixture was loaded on the membrane. DOC Gel 2,000 gel imaging system (Bio-Rad, Hercules, CA, USA) was used to calculate the integral optical density of target protein and compare it with β-actin.

### Statistical analysis

All data were expressed as the mean ± standard deviation (*x* ± SD). Hemodynamic indexes were compared using a one-way analysis of variance with repeated measures (repeated measures ANOVA). Student’s *t*-test and ANOVA were calculated with SPSS 17.0 statistical software. *P* ≤ 0.05 was considered statistically significant.

## Results

### The effects of paclitaxel and colchicine in disrupting the microtubular structure of cardiomyocytes

**Figure 1:**
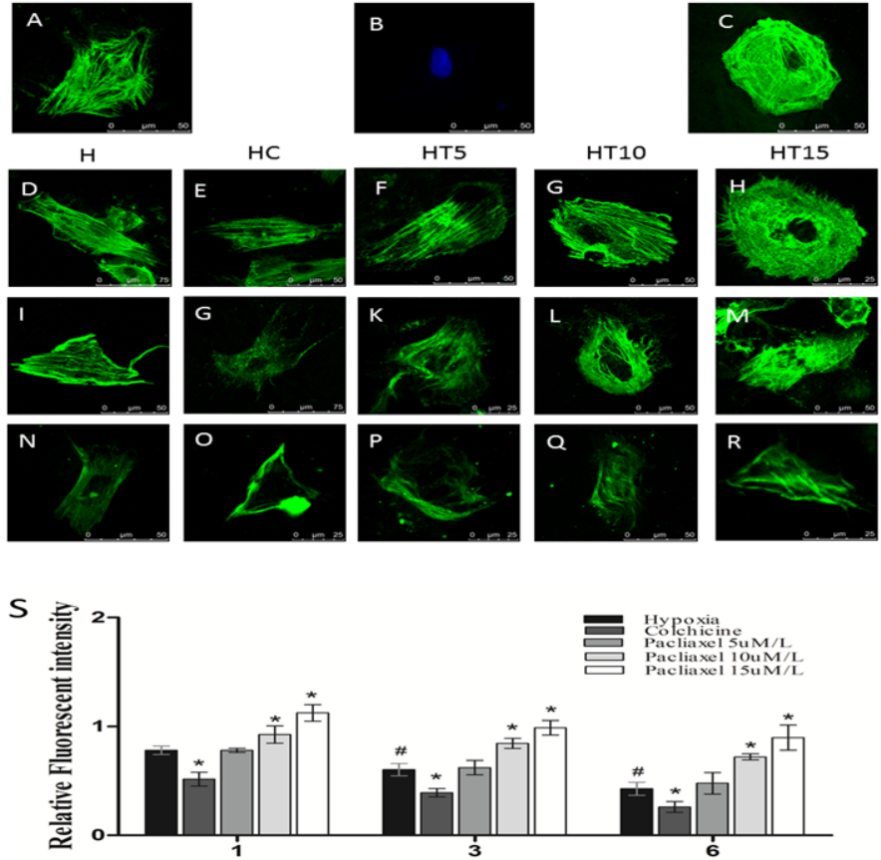
The influence of paclitaxel and colchicine on microtubular structure in cardiomyocytes exposed to different durations of hypoxia. Note: (A) positive control; (B) negative control; (C) microtubule fluorescence staining with an anti-rat-microtubulin antibody under normal culture conditions (normoxic cardiomyocytes), α-microtubulin (green); (D–R) Fluorescence of the microtubular structure after 1, 3, and 6 h of hypoxia; (H) hypoxia injury group, HC, colchicine group (10 µmol/L group); HT5, low-dose paclitaxel group (5 µmol/L group), HT10, mid-dose paclitaxel group (10 µmol/L group), HT15, high-dose paclitaxel group (15 µmol/L group). S, Nine cardiomyocytes were selected randomly from each group to detect the relative fluorescence intensity; the fluorescence intensity of the microtubules from normally cultivated cells was set to a value of 1.0, and experimental values were normalized with respect to the normoxic value. Paclitaxel at a concentration of 10 µmol/L could prevent the hypoxia-induced injury of microtubular structure. Values indicate means and SD, *n* = 9. *P < 0.05 vs. H group.

Under normoxic conditions, cultivated cardiomyocytes exhibited a complete reticular microtubular structure and clearly visible microtubules around membranes (Figs. 1A–1C). As the duration of hypoxia injury increased, the microtubular structure suffered increasingly serious damage, and the fluorescence intensity gradually weakened (Figs. 1D–1F, H group). The HC group acquired a disordered, broken, and unevenly distributed microtubular structure after 1 h of hypoxia injury. As the duration of hypoxia injury lengthened, the extent of injury to the microtubular structure was exacerbated (Figs. 1D–1F, HC group). In the HT group, treatment with increasing paclitaxel concentrations caused the fluorescence intensity of microtubulin to gradually increase relative to hypoxia alone. After 1 h or 3 h of hypoxia, the fluorescence intensity of microtubulin in the group treated with 5 µmol/L paclitaxel did not differ significantly from that of the hypoxia group; however, after 6 h of hypoxia injury, the microtubulin fluorescence intensity in the cardiomyocytes treated with all other concentrations and times was significantly higher than that in the hypoxia group.

The low dose of paclitaxel (5 µmol/L) could slightly prevent the microtubule injury caused by hypoxia, and the stabilization function increased with the rising paclitaxel concentration. For instance, after treatment with 15 µmol/L paclitaxel, the microtubule fluorescence intensity increased remarkably; however, although the fluorescence intensity of the microtubules in the paclitaxel-treated group was relatively high, it appeared to change the structure of the microtubules, causing uneven sheet distribution within the endochylema and patchy aggregations of microtubulin in the cytoplasm (Figs. 1D–1F, HT15 group). In the groups cultured under normal conditions, the changes in microtubular structure that occurred with different concentrations of paclitaxel were similar to the above changes; however, the microtubular structure was clearer than that in the hypoxia group and exhibited a higher fluorescence intensity.

### The influence of hypoxia preconditioning on microtubular structure

**Figure 2:**
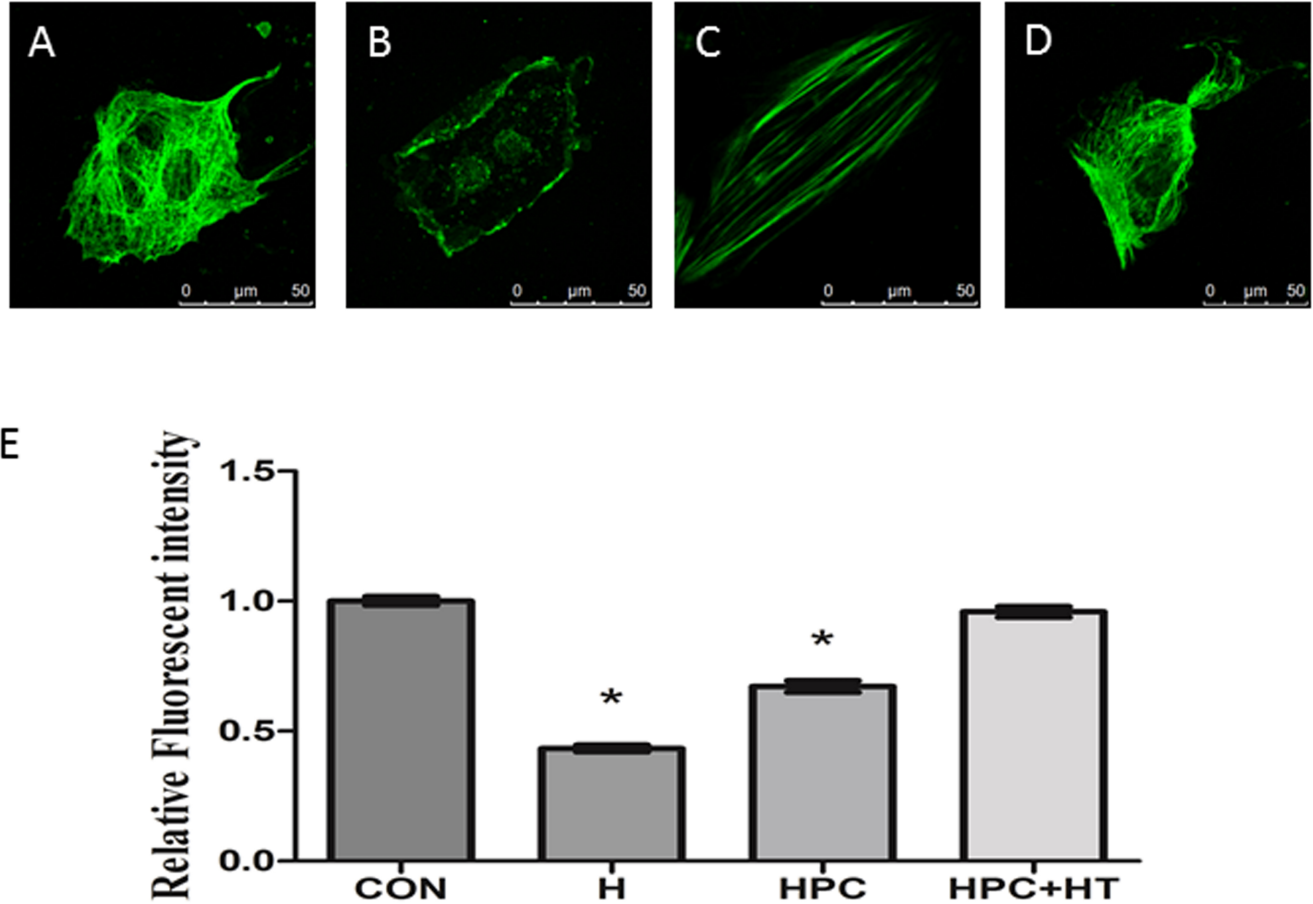
The influence of hypoxia preconditioning on microtubular structure injury in cardiomyocytes. (A) Cardiomyocytes in normal culture; (B) Cardiomyocytes exposed to hypoxia for 6 h; (C) cardiomyocytes exposed to 2-h hypoxia, 2-h reoxygenation and then 6 h of hypoxia; (D) Cardiomyocytes were treated with 10 µmol/L paclitaxel for 12 h, then exposed to hypoxia preconditioning and hypoxia injury; (E) The fluorescence intensities of microtubular structures in all intervention groups were analyzed using Image-Pro Plus software. Nine cardiomyocytes were randomly selected to detect the relative fluorescence value of the microtubular structure. The fluorescence intensity in the normal group was set to a value of 1.0. Values indicate the means and SD; *n* = 9. *P < 0.05 vs. H group.

Hypoxia preconditioning has a protective effect on cardiomyocytes; however, it is not yet known what changes occur in microtubules during this process. First, it was observed that the microtubular structure in normal cardiomyocytes (control group) appeared to have a grid arrangement, with uniform staining and clearly visible microtubules around the membrane (Fig. 2A). After cardiomyocytes were exposed to hypoxia for 6 h, the microtubular structure became indistinct, the grid structure was extensively damaged and unstained vacuoles were observed in some areas (Fig. 2B). The fluorescence intensity was reduced relative to that in the normal culture group (*p* < 0.05).

The injury to the microtubular structure was less than that observed after hypoxia injury alone, featuring less obvious uneven staining, fewer broken structures, and no contracted cells. The fluorescence intensity was significantly higher than that in the hypoxia injury group (Fig. 2C). This showed that hypoxia preconditioning could enhance the stability of the microtubular structure. After treatment with the microtubule stabilizer paclitaxel (10 µmol/L) in combination with hypoxia preconditioning, the microtubular structure of cardiomyocytes was complete, exhibiting continuous and uniform staining similar to that observed for normal microtubules, with the fluorescence intensity apparently higher than that in the hypoxia injury group (Fig. 2D). This revealed that hypoxia preconditioning combined with paclitaxel (10 µmol/L) prevented microtubular structure injury in cardiomyocytes better than treatment with paclitaxel or preconditioning alone.

### The effects of the changes in microtubular reticular structure on HIF-1α endonuclear localization in hypoxic cardiomyocytes

**Figure 3:**
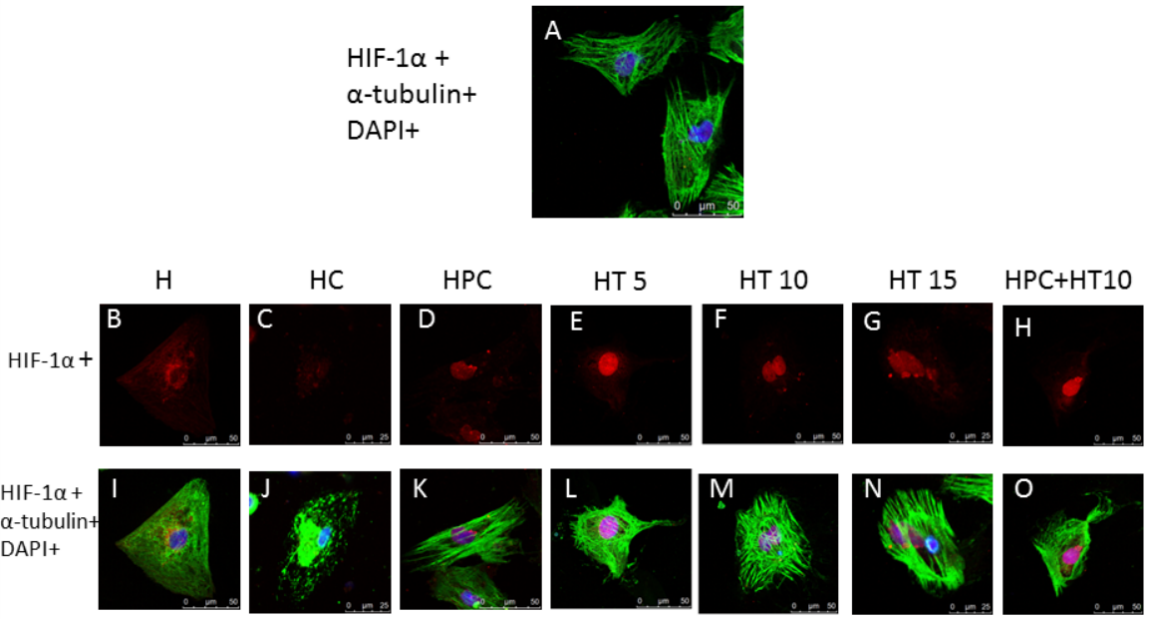
The influence of the changes in the microtubular structure of cardiomyocytes on HIF-1α endonuclear translocation. Note: (A) cells were labeled with α-tubulin and HIF-1α and then visualized with secondary antibodies conjugated to FITC (green) and tetramethyl-rhodamine isothiocyanate (TRITC, red), and then the nuclei were stained with 4′, 6-diamidino-2-phenylindole (DAPI, blue); (B, I) cardiomyocytes exposed to hypoxia for 6-h hypoxia; (C, J) colchicine group (HC group); (D, K) cardiomyocytes exposed to 2-h hypoxia, 2-h reoxygenation and then 6 h of hypoxia (HPC group); (E, L) cardiomyocytes were treated with 5 µmol/L paclitaxel for 12 h and hypoxia injury (HT5 group); (F, M) cardiomyocytes were treated with 10 µmol/L paclitaxel for 12 h and hypoxia injury (HT10 group); (G, N) cardiomyocytes were treated with 10 µmol/L paclitaxel for 12 h and hypoxia injury (HT15 group); (H, O) cardiomyocytes were treated with 10 µmol/L paclitaxel for 12 h, then exposed to hypoxia preconditioning and hypoxia injury (HPC + HT10).

The goal of these experiments was to identify the role of microtubules in the HIF-1α nuclear entry process by altering microtubular structure with microtubule stabilizing agents and depolymerizing agents. Immunofluorescence staining of the microtubules, α-tubulin and HIF-1α in normally cultured cells revealed that the microtubules appeared in a clear grid, with a uniform distribution in the cytoplasm and clear microtubular structure near the cytomembrane; moreover, low HIF-1α expression was observed (Fig. 3A).

After the cells were exposed to hypoxia for 6 h, the microtubules were indistinct and exhibited a disorganized microtubular structure; HIF-1α was expressed in the cytoplasm and nucleus (Fig. 3B). Treatment with a microtubule depolymerizing agent and hypoxia injury caused microtubules to become indistinct, unclear, broken, and severely damaged. HIF-1α appeared to be restricted around the nucleus and was rarely localized within the nucleus, illustrating that the damaged microtubular structure affected HIF-1α accumulation within the nucleus (Fig. 3B). In the colchicine group, HIF-1α localized around the nucleus (Fig. 3C). Hypoxia preconditioning attenuated the hypoxia-induced injury to the microtubular structure, and extensive HIF-1α staining appeared in the nucleus and was rarely present within the cytoplasm, revealing that hypoxia preconditioning could stabilize microtubular structure and promote HIF-1α localization in the nucleus (Fig. 3D).

Treating cardiomyocytes with different concentrations (5 µmol/L, 10 µmol/L, and 15 µmol/L) of the microtubule stabilizing agent paclitaxel before hypoxia injury revealed that maximum HIF-1α localization in the nucleus occurred in the 10 µmol/L paclitaxel group (Fig. 3F). The group treated with 15 µmol/L paclitaxel exhibited higher fluorescence intensity, but the localization pattern changed to a sheet-shaped structure. Although amount of HIF-1α localized in the nucleus was relatively high, a large amount of HIF-1α aggregated around, but did not enter, the nucleus, which revealed that a high dose of paclitaxel could cause the microtubular structure to become disordered, thus affecting the HIF-1α nuclear translocation process (Fig. 3G). HIF-1α staining in the Hypoxia preconditioning and 10 µmol/L paclitaxel-treated cells showed relatively intense staining. The staining, while more intense around the nucleus (Fig. 3H).

### The effects of microtubule reticular changes on HIF-1α protein content

**Figure 4:**
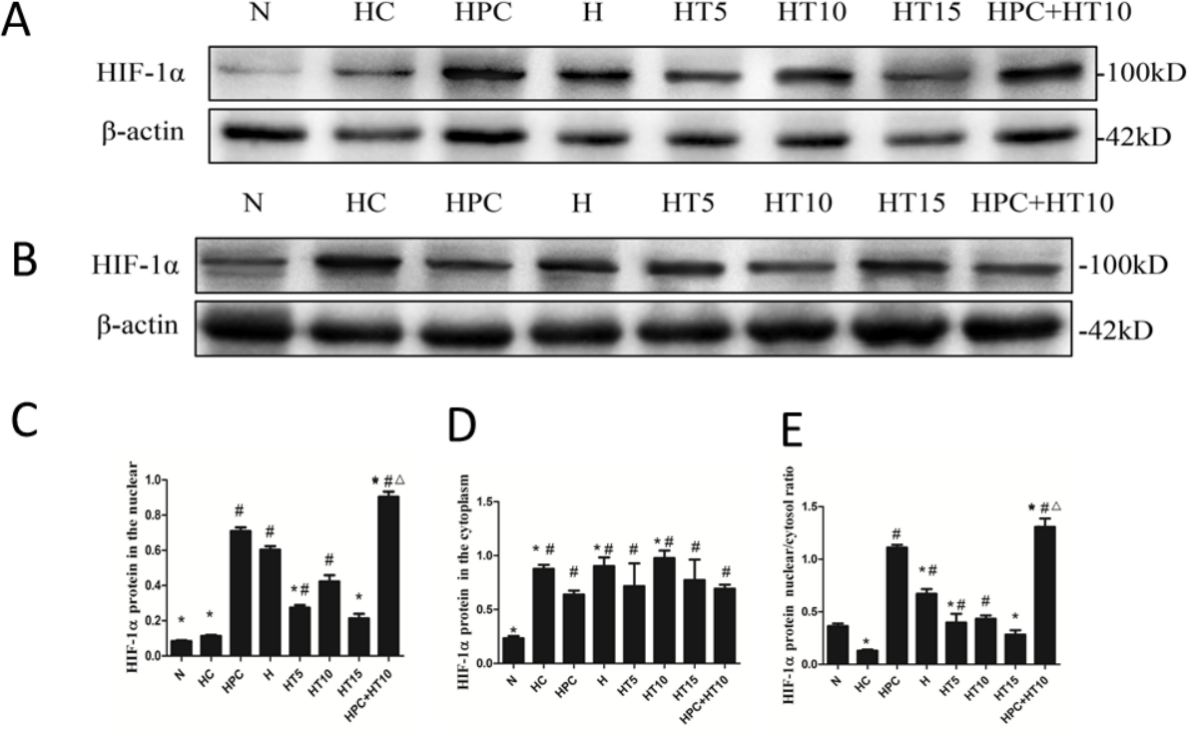
Effect of microtubule reticular changes on HIF-1 protein content. (A) Expressions of HIF-1α in the nucleus. (B) Expressions of HIF-1α in the cytoplasm. (C) Relative protein levels for HIF-1α quantified in the nucleus. (D) Relative protein levels for HIF-1α quantified in the cytoplasm. (E) Ratio of nucleus HIF-1α to cytoplasm HIF-1α. The data showed that after hypoxia pre-treatment and/or taxol intervention, nucleus HIF-1α expression was increased while cytoplasmic HIF-1α expression was declined incardiomyocytes. After colchicine intervention, the nuclear/cytoplasmic ratio of HIF-1α was lower, suggesting that after colchicine injured microtubules, HIF-1α protein gathered in the cytoplasm instead of entering into the nucleus. #*P* < 0.05 vs. N group; *P < 0.05 vs. HPC group. △*P* < 0.05 vs. HT 10 group.

This study detected HIF-1α protein in nucleus and cytoplasm, respectively, and calculated the ratio of nucleus to cytoplasm HIF-1a protein. We found that it was significantly higher in the HPC group, H group, HT5 group, HT10 group and the HPC + HT10 group than that of the H group, suggesting that after hypoxia pre-treatment and/or taxol intervention, nucleus HIF-1a expression was increased while cytoplasmic HIF-1α expression was declined in cardiomyocytes. The ratio of nucleus to cytoplasmic HIF-1α was significantly higher in the HPC + HT10 group than that of HPC group and HT10 group, suggesting that hypoxia pre-treatment combined with middle concentration of taxol can promote HIF-1α to enter into nucleus. The ratio of nucleus to cytoplasmic HIF-1α expression was significantly lower in HC group than those of the HPC group, H group, HT5 group, HT10 group and the HPC + HT10 group, suggesting that after colchicine injured microtubules, a large amount of HIF-1a protein gathered in the cytoplasm while a small amount entered into the nucleus (Figs. 4A–4E).

### The influence of hypoxia preconditioning combined with microtubule stabilization on cardiomyocyte injury and energy generation

The previous sections indicated that hypoxia preconditioning can stabilize the microtubular structure, which plays a key role in HIF-1 nuclear translocation. Therefore, this study sought to verify whether stabilization of the microtubular structure can enhance the myocardial protective effect. First, it was discovered that LDH content was significantly higher after hypoxia injury. LDH content was significantly reduced by hypoxia preconditioning and paclitaxel, and the lowest LDH content was observed after treatment with hypoxia preconditioning combined with paclitaxel intervention, with the LDH content in this group being significantly lower than that in the hypoxia injury group (Table 1).

**Table 1:** The influence of hypoxia preconditioning combined with microtubule stabilization on cardiomyocyte injury and energy generation. The LDH content of cardiomyocytes (IU/g protein, mean ± SD) was detected after treatment with different interventions for 12 h. The PFK (IU/g protein, mean ± SD), ATP, and ADP (mg/g protein, mean ± SD) content. E–F, The PK and HK content (IU/g protein, mean ± SD). The above data showed that paclitaxel could enhance the myocardial protective effect of hypoxia preconditioning.

	LDH	ADP	ATP	PK	HK	PFK	ROS
N	262.3± 88.80^a^^,^^b^	4.75 ± 0.621^a^^,^^b^	26 ± 1.12^a^^,^^b^	13.41 ± 1.50^a^^,^^b^	15.33 ± 1.30^a^^,^^b^	21 ± 1.41^a^^,^^b^	2.39 ± 0.45^a^^,^^b^
H	1663 ± 155.8^b^^,^^c^	14.91 ± 1.08^b^^,^^c^	11.58 ± 0.99^b^^,^^c^	479.5 ± 39.64^b^^,^^c^	138.9 ± 18.90^c^	175.7 ± 11.39^b^^,^^c^	7.70 ± 0.68^b^^,^^c^
HPC	759.6 ± 132.7^a^^,^^c^	12.41 ± 0.79^a^^,^^c^	18.41 ± 1.16^a^^,^^c^	687.7 ± 55.30^a^^,^^c^	133.7 ± 12.54^c^	215.4 ± 9.74^c^	3.90 ± 0.31^a^
HT5	1088.9 ± 122.3^a^^,^^b^^,^^c^	12.33 ± 1.30^a^^,^^c^	12.83 ± 1.02^a^^,^^c^	803.2 ± 51.28^a^^,^^c^	141.3 ± 13.03^c^	154.5 ± 11.42^b^^,^^c^	6.25 ± 0.23^a^^,^^b^^,^^c^
HT10	956.0 ± 135.5^a^^,^^c^	13.50 ± 0.52^a^^,^^c^	15.41 ± 0.90^a^^,^^c^	879.6 ± 65.79^a^^,^^c^	152.8 ± 6.16^c^	181.2 ± 9.81^b^^,^^c^	5.10 ± 0.33^a^^,^^b^^,^^c^
HT15	1091.5 ± 162.8^a^^,^^c^	15.83 ± 1.58^a^^,^^b^^,^^c^	10.50 ± 1.50^a^^,^^b^^,^^c^	683.3 ± 87.68^a^^,^^c^	133.9 ± 12.39^c^	139.4 ± 16.78^b^^,^^c^	6.25 ± 0.17^a^^,^^b^^,^^c^
HPC + HT10	393.7 ± 130.9^a^^,^^b^^,^^c^^,^^d^	5.83 ± 0.71^a^^,^^b^^,^^c^^,^^d^	23.16 ± 1.52^a^^,^^b^^,^^c^^,^^d^	1106.8 ± 85.64^a^^,^^b^^,^^c^^,^^d^	205.1 ± 12.11^a^^,^^b^^,^^c^^,^^d^	263.8 ± 13.82^a^^,^^b^^,^^c^^,^^d^	3.20 ± 0.49^a^^,^^b^^,^^c^^,^^d^

**Notes.**

a Significantly different from the H group.

b Significantly different from the HPC group.

c Significantly different from the N group.

d Significantly different from the HT10 group.

The trends in the data were very similar for PK, HK, and PFK. The cellular PK, HK, and PFK content increased significantly after hypoxia injury of cardiomyocytes. Thus, the cellular PK, HK, and PFK content in the hypoxia preconditioning combined with the paclitaxel group not only was the highest but also was significantly higher than that in all the other groups (Table 1).

The ATP level indicates the ability of cardiomyocytes to generate energy. The ATP content in all the hypoxia injury groups decreased gradually, whereas the ATP content in the groups treated with hypoxia preconditioning combined with paclitaxel intervention was higher than that in the hypoxia preconditioning group and the paclitaxel group. In contrast to the changes in ATP content, the ADP content in all the hypoxia injury groups was significantly elevated, whereas the level of ADP rose markedly in the hypoxia preconditioning combined with the paclitaxel intervention group (Table 1).

Oxygen free radicals and lipid peroxidation are the initiation factors of myocardial damage. We found that the content of ROS was decreased after hypoxic preconditioning, which indicated that hypoxic preconditioning could effectively reduce the amount of ROS produced by hypoxic injury. After the stabilization of the microtubule structure, ROS production was also reduced, which might also play a role in myocardial protection. (Table 1).

## Discussion

We first consolidated altered microtubular structures of cardiomyocytes with the microtubule stabilizing agent paclitaxel and the microtubule-depolymerizing agent colchicine and then observed their effects on microtubular structures in cultured cardiomyocytes. Colchicine can cause the dissociation of microtubules and inhibit the polymerization of free tubulin (Pimperl et al., 2016), while paclitaxel can stabilize the microtubule structure to achieve aggregation and can antagonize the depolymerizing effect of colchicine (Ehler & Perriard, 2000). This study found that hypoxia could depolymerize myocardial microtubules and damage the microtubule reticular structure; This study set up different hypoxia injury times (1 h, 3 h, 6 h). As the time of hypoxia injury gradually prolonged, the damage of microtubule structure became more and more serious, and the fluorescence value of tubulin decreased gradually.

Microtubule depolymerizing agents can significantly increase the damage to the microtubule structure and cause the reticular distribution of microtubules to disappear. An appropriate concentration (10 µM) of paclitaxel can better maintain the reticular structure of microtubules in the early hypoxic stage and improve the damage to the myocardial microtubule reticular structure caused by hypoxia. Although high doses (15 µM) of paclitaxel can promote tubulin polymerization, excessive polymerization can change the normal reticular structure and destroy the dynamic balance of microtubules under physiological conditions. This study found that a period of time of early stage (1–3 h) after hypoxia, 10 µM paclitaxel can maintain normal microtubule structure of cardiomyocytes and as such, cardiomyocytes damage is minimum. However, this protective effect gradually diminished with the aggravation of hypoxia. However, the microtubule stabilization function of 5 µM paclitaxel is slightly weaker, which can resist microtubule disruption due to hypoxia only in the early stage of hypoxia when microtubule damage is weaker, which can also slow cardiomyocytes injury.

Hypoxia preconditioning refers to the exposure of cells to a repeated and brief hypoxic stimulus that can trigger and mobilize the intrinsic self-protection ability of cells to prevent a variety of subsequent physiological and pathological changes (Dou et al., 2016; Selvaraj et al., 2016; Xu et al., 2016). In 1995, Webster et al. observed that transient hypoxia preconditioning can protect rat cardiomyocytes from long-term anoxic injury, providing evidence of the feasibility of preconditioning research at the cellular level. The mechanism underlying hypoxia preconditioning may involve various factors, such as the expression of hypoxia-responsive genes, vascular remodeling, changes in energy metabolism, and resistance to oxidative stress, among others (Webster, 1997; Zhang et al., 2016). Microtubules, as an important cellular structure in cardiomyocytes, play a vital role in cardiomyocyte function. In subsequent experiments, we explored the influence of hypoxia preconditioning on the microtubule structure in cardiomyocytes. We observed more complete microtubule structure in the hypoxia preconditioning group than in the simple hypoxia injury group. In addition, a comparison of the relative fluorescence value of tubulin also showed that the fluorescence intensity of tubulin in the hypoxia preconditioning group was higher than that in the hypoxia injury group. These results suggested that hypoxic pretreatment can stabilize the structure of microtubules in cardiomyocytes.

Next, we observed how changes in the microtubule structure influence the nuclear translocation of HIF-1α. Our early results showed that HIF-1α is one of the important nuclear transcription factors that initiate endogenous myocardial protection (Zheng et al., 2015). Generally, HIF-1α protein was rapidly degraded under normal oxygen condition via ubiquitin-proteasome system mediated by protein von HippelLindau (PVHL), while anoxic environment prevented this degradation, thus inducing the accumulation of HIF-1α; accumulated HIF-1α in plasma will enter nucleus and combine with β subunit, triggering down-stream hypoxia adaptive genes, which can enhance the adaptability of myocardium to hypoxia (Dou et al., 2016). The focus of this article is to uncover the influence factors for the expression of HIF-1α in nucleus from cytoplasm under hypoxia state. Microtubules run through entire cardiomyocytes in a double helix structure, with one end connecting with nucleus and the other end in cytoplasm (Xiaodong et al., 2015). The distribution characteristics of microtubules play an important role in maintaining the normal form and position of nucleus (Hasan et al., 2006). Microtubules also have important functions of information transfer and material transport (Ehler & Perriard, 2000). The information molecule in cardiomyocyte cytoplasm is transferred through sarcolemma, microtubules, and nucleus (Xiaodong et al., 2015). Therefore, we are naturally interested in the potential relationship between HIF-1α and the cardiomyocyte microtubule. There is no direct evidence about whether the changes of the microtubule structure of cardiomyocytes can modulate the dislocation of HIF-1α in nucleus.

To test this, Colchicine can cause the dissociation of microtubules and inhibit the polymerization of free tubulin (Pimperl et al., 2016), while paclitaxel can stabilize the microtubule structure to achieve aggregation and can antagonize the depolymerizing effect of colchicine (Escuin, Kline & Giannakakou, 2005). Microtubule depolymerizing agents can significantly increase the damage to the microtubule structure and cause the reticular distribution of microtubules to disappear. An appropriate concentration (10 µM) of paclitaxel can better maintain the reticular structure of microtubules in the early hypoxic stage and improve the damage to the myocardial microtubule reticular structure caused by hypoxia. Although high doses (15 µM) of paclitaxel can promote tubulin polymerization, excessive polymerization can change the normal reticular structure and destroy the dynamic balance of microtubules under physiological conditions. This study found that blindly pursuing myocardial microtubule stability cannot protect the cardiomyocytes; instead, a certain concentration of paclitaxel can protect cardiomyocytes.

We found that under anoxic conditions, the stability of the microtubule network structure was consistent with the increased expression and nuclear entry of HIF-1α and increased protein content. Further disruption or elimination of the microtubule network structure may occur due to hypoxia or depolymerizing agents, or microtubules may excessively polymerize due to high-dose stabilizer and an uneven distribution in the cytoplasm; these changes can all decrease the expression and nuclear entry of HIF-1α. The expression of HIF-1α directly affected the anti-hypoxia ability of cardiomyocytes. Consequently, the microtubule structure directly participated in the nuclear translocation of HIF-1α. In the third part of the results, we found that the hypoxia preconditioning was able to stabilize the microtubule structure, which further elucidated the internal mechanism of hypoxia preconditioning in myocardial protection.

This research evaluated the effects of the microtubule stabilizing agent on hypoxic preconditioning effects by measuring changes in the generation of energy in cardiomyocytes. ADP and ATP content can directly reflect the generation of energy by cardiomyocytes. The results showed that the generation of ATP decreased significantly, while ADP levels increased significantly in response to hypoxia. More ATP was generated after hypoxia preconditioning combined with paclitaxel intervention than after hypoxia injury or hypoxia preconditioning, suggesting paclitaxel combined with hypoxia preconditioning increased the generation of energy in cardiomyocytes. In addition, we detected the HK, PFK, and PK levels; all of these kinases are key rate-limiting enzymes in the glycolytic pathway in cardiomyocytes and can indirectly reflect the condition of cardiomyocyte energy metabolism (Volker, Reinitz & Knull, 1995). We found that the HK, PFK, and PK levels increased after hypoxia injury, and the glycolytic activity of cardiomyocytes, was significantly enhanced in early hypoxia. In the early period of hypoxia, the activity of a key enzyme in the glycolytic pathway of cardiomyocytes increased significantly in response to the appropriate concentration of the microtubule stabilizer, and this level was higher than the activity of this enzyme in the simple hypoxia group. Among the groups, the activity of the key glycolytic enzyme was the highest in the hypoxia preconditioning and paclitaxel intervention group. We reasoned that if hypoxia damaged the myocardial microtubule structure, then the structure and function of the mitochondria are damaged, and the amount of energy supplied by oxidative phosphorylation is inhibited. We also reasoned that appropriately stabilizing the microtubule structure in the case of hypoxia preconditioning can improve myocardial energy metabolism.

Finally, we attempted to clarify whether the microtubule stabilizer can enhance hypoxia preconditioning. The LDH level in the culture medium of cardiomyocytes is a sensitive indicator of cardiomyocyte damage. We found that the LDH level increased significantly in the culture medium of the simple hypoxia injury group, and the LDH level decreased after hypoxia preconditioning. The LDH level in the groups treated with the microtubule stabilizer and hypoxia preconditioning was lower than that in the other groups. These results suggested that stabilizing the microtubule structure can enhance the protective effect of hypoxia preconditioning on cardiomyocytes.

In addition, this study also observed the reactive oxygen species (ROS) changes during hypoxia. ROS, such as superoxide anion, hydroxyl radicals (OH−), peroxyl, and oxygen-derived nonradical species (e.g., hydrogen peroxide (H_2_O_2_), are a group of oxygen molecules with high activity (Chetram & Hinton, 2013). During myocardial cell hypoxia, ROS are increased, resulting in the inactivation of many important enzymes, including the lipid membrane Na^+^/Ca^2+^ pump, creatine kinase, and mitochondria dehydrogenase, eventually leading to myocardial cell apoptosis and other effects. This study found that the hypoxia preconditioning of cardiomyocytes and a stable microtubule structure can significantly reduce ROS levels. Previous reports showed that PO2 cycling preconditioning in mice and found a significant reduction in the ROS produced by the mouse diaphragm and a consequent decrease in the fatigue of the diaphragm (Zuo et al., 2014). Zuo et al. (2015) scholars found in another study that PO2 cycling preconditioning can protect the diaphragm, and the mechanism involves promotion of the opening of mitochondrial ATP-sensitive potassium channels and the closure of mPTP channels, with the ROS/Akt/ERK signaling pathway participating in this regulation. This study only used ROS as an indicator to reflect myocardial hypoxia injury, and the authors will evaluate the internal relationship between microtubules and ROS in further research.

In conclusion, the microtubules of cardiomyocytes may be involved in the process of HIF-1α endonuclear aggregation under the condition of hypoxia preconditioning, which enhances the anti-hypoxia ability of cardiomyocytes.

##  Supplemental Information

10.7717/peerj.3662/supp-1 Data S1 Raw data

## Additional Information and Declarations

### Data Availability

The following information was supplied regarding data availability: The raw data has been supplied as Data S1.

## References

[ref-1] Chetram MA, Hinton CV (2013). ROS-mediated regulation of CXCR4 in cancer. Frontiers in Biology.

[ref-2] Dou MY, Wu H, Zhu HJ, Jin SY, Zhang Y, He SF (2016). Remifentanil preconditioning protects rat cardiomyocytes against hypoxia-reoxygenation injury via delta-opioid receptor mediated activation of PI3K/Akt and ERK pathways. European Journal of Pharmacology.

[ref-3] Duyndam MC, Van Berkel MP, Dorsman JC, Rockx DA, Pinedo HM, Boven E (2007). Cisplatin and doxorubicin repress Vascular Endothelial Growth Factor expression and differentially down-regulate Hypoxia-inducible Factor I activity in human ovarian cancer cells. Biochemical Pharmacology.

[ref-4] Ehler E, Perriard JC (2000). Cardiomyocyte cytoskeleton and myofibrillogenesis in healthy and diseased heart. Heart Failure Reviews.

[ref-5] Escuin D, Kline ER, Giannakakou P (2005). Both microtubule-stabilizing and microtubule-destabilizing drugs inhibit hypoxia-inducible factor-1alpha accumulation and activity by disrupting microtubule function. Cancer Research.

[ref-6] Hasan MR, Koikawa S, Kotani S, Miyamoto S, Nakagawa H (2006). Ferritin forms dynamic oligomers to associate with microtubules *in vivo*: implication for the role of microtubules in iron metabolism. Experimental Cell Research.

[ref-7] Jain K, Suryakumar G, Prasad R, Ganju L (2013). Upregulation of cytoprotective defense mechanisms and hypoxia-responsive proteins imparts tolerance to acute hypobaric hypoxia. High Altitude Medicine & Biology.

[ref-8] Jiang X, Zhang D, Zhang H, Huang Y, Teng M (2015). Role of Ran-regulated nuclear-cytoplasmic trafficking of pVHL in the regulation of microtubular stability-mediated HIF-1alpha in hypoxic cardiomyocytes. Scientific Reports.

[ref-9] Kim HA, Rhim T, Lee M (2011). Regulatory systems for hypoxia-inducible gene expression in ischemic heart disease gene therapy. Advanced Drug Delivery Reviews.

[ref-10] Kumarapeli AR, Wang X (2004). Genetic modification of the heart: chaperones and the cytoskeleton. Journal of Molecular and Cellular Cardiology.

[ref-11] Malhotra R, Tyson DG, Sone H, Aoki K, Kumagai AK, Brosius III FC (2002). Glucose uptake and adenoviral mediated GLUT1 infection decrease hypoxia-induced HIF-1alpha levels in cardiac myocytes. Journal of Molecular and Cellular Cardiology.

[ref-12] Miragoli M, Sanchez-Alonso JL, Bhargava A, Wright PT, Sikkel M, Schobesberger S, Diakonov I, Novak P, Castaldi A, Cattaneo P, Lyon AR, Lab MJ, Gorelik J (2016). Microtubule-dependent mitochondria alignment regulates calcium release in response to nanomechanical stimulus in heart myocytes. Cell Reports.

[ref-13] Murry CE, Jennings RB, Reimer KA (1986). Preconditioning with ischemia: a delay of lethal cell injury in ischemic myocardium. Circulation.

[ref-14] Napolitano MJ, Shain DH (2005). Quantitating adenylate nucleotides in diverse organisms. Journal of Biochemical and Biophysical Methods.

[ref-15] Ong SG, Hausenloy DJ (2012). Hypoxia-inducible factor as a therapeutic target for cardioprotection. Pharmacology and Therapeutics.

[ref-16] Pimperl A, Schulte T, Muhlbacher A, Rosenmoller M, Busse R, Groene O, Rodriguez HP, Hildebrandt H (2016). Evaluating the impact of an accountable care organization on population health: the quasi-experimental design of the german gesundes kinzigtal. Population Health Management.

[ref-17] Robison P, Prosser BL (2017). Microtubule mechanics in the working myocyte. Journal of Physiology.

[ref-18] Rossini R, Musumeci G, Visconti LO, Bramucci E, Castiglioni B, De Servi S, Lettieri C, Lettino M, Piccaluga E, Savonitto S, Trabattoni D, Capodanno D, Buffoli F, Parolari A, Dionigi G, Boni L, Biglioli F, Valdatta L, Droghetti A, Bozzani A, Setacci C, Ravelli P, Crescini C, Staurenghi G, Scarone P, Francetti L, D’Angelo F, Gadda F, Comel A, Salvi L, Lorini L, Antonelli M, Bovenzi F, Cremonesi A, Angiolillo DJ, Guagliumi G (2014). Perioperative management of antiplatelet therapy in patients with coronary stents undergoing cardiac and non-cardiac surgery: a consensus document from Italian cardiological, surgical and anaesthesiological societies. EuroIntervention.

[ref-19] Selvaraj UM, Ortega SB, Hu R, Gilchrist R, Kong X, Partin A, Plautz EJ, Klein RS, Gidday JM, Stowe AM (2016). Preconditioning-induced CXCL12 upregulation minimizes leukocyte infiltration after stroke in ischemia-tolerant mice. Journal of Cerebral Blood Flow and Metabolism.

[ref-20] Shohet RV, Garcia JA (2007). Keeping the engine primed: HIF factors as key regulators of cardiac metabolism and angiogenesis during ischemia. Journal of Molecular Medicine.

[ref-21] Teng M, Jiang XP, Zhang Q, Zhang JP, Zhang DX, Liang GP, Huang YS (2012). Microtubular stability affects pVHL-mediated regulation of HIF-1alpha via the p38/MAPK pathway in hypoxic cardiomyocytes. PLOS ONE.

[ref-22] Tuomainen T, Tavi P (2017). The role of cardiac energy metabolism in cardiac hypertrophy and failure. Experimental Cell Research.

[ref-23] Volker KW, Reinitz CA, Knull HR (1995). Glycolytic enzymes and assembly of microtubule networks. Comparative Biochemistry and Physiology - Part B.

[ref-24] Webster DR (1997). Regulation of post-translationally modified microtubule populations during neonatal cardiac development. Journal of Molecular and Cellular Cardiology.

[ref-25] Xiaodong L, Yongming D, Lingfei L, Qiong Z, Yuesheng H (2015). Effects of microtubule depolymerization on spontaneous beating and action potential of cardiac myocytes in rats and its mechanism. Zhonghua Shao Shang Za Zhi.

[ref-26] Xu R, Sun Y, Chen Z, Yao Y, Ma G (2016). Hypoxic preconditioning inhibits hypoxia-induced apoptosis of cardiac progenitor cells via the PI3K/Akt-DNMT1-p53 pathway. Scientific Reports.

[ref-27] Yu J, Wu J, Xie P, Maimaitili Y, Wang J, Xia Z, Gao F, Zhang X, Zheng H (2016). Sevoflurane postconditioning attenuates cardiomyocyte hypoxia/reoxygenation injury via restoring mitochondrial morphology. PeerJ.

[ref-28] Zhang R, Li L, Yuan L, Zhao M (2016). Hypoxic preconditioning protects cardiomyocytes against hypoxia/reoxygenation-induced cell apoptosis via sphingosine kinase 2 and FAK/AKT pathway. Experimental and Molecular Pathology.

[ref-29] Zheng H, Guo H, Hong Y, Zheng F, Wang J (2015). The effects of age and resveratrol on the hypoxic preconditioning protection against hypoxia-reperfusion injury: studies in rat hearts and human cardiomyocytes. European Journal of Cardio-Thoracic Surgery.

[ref-30] Zuo L, Diaz PT, Chien MT, Roberts WJ, Kishek J, Best TM, Wagner PD (2014). PO2 cycling reduces diaphragm fatigue by attenuating ROS formation. PLOS ONE.

[ref-31] Zuo L, Pannell BK, Re AT, Best TM, Wagner PD (2015). Po2 cycling protects diaphragm function during reoxygenation via ROS, Akt, ERK, and mitochondrial channels. American Journal of Physiology. Cell Physiology.

